# MicroRNA-7 Regulates Migration and Chemoresistance in Non-Hodgkin Lymphoma Cells Through Regulation of KLF4 and YY1

**DOI:** 10.3389/fonc.2020.588893

**Published:** 2020-10-27

**Authors:** Mario Morales-Martinez, Gabriel G. Vega, Natividad Neri, M. J Nambo, Isabel Alvarado, Ivonne Cuadra, M. A. Duran-Padilla, Sara Huerta-Yepez, Mario I. Vega

**Affiliations:** ^1^Molecular Signal Pathway in Cancer Laboratory, Unidad de Investigación Medica en Enfermedades Oncologicas (UIMEO), Oncology Hospital, Siglo XXI National Medical Center, Instituto Méxicano del Seguro Social (IMSS), Mexico City, Mexico; ^2^Unidad de Posgrado, Facultad de Medicina, Universidad Nacional Autónoma de México, Mexico City, Mexico; ^3^Department of Hematology, Oncology Hospital, National Medical Center, IMSS, Mexico City, Mexico; ^4^Servicio de Anatomía Patológica, Hospital de Oncología, Centro Médico Nacional Siglo XXI, IMSS, Mexico City, Mexico; ^5^Servicio de Patología, Hospital General de México “Eduardo Liceaga”, Facultad de Medicina de la UNAM, Mexico City, Mexico; ^6^Unidad de Investigación en Enfermedades Oncológicas, Hospital Infantil de México Federico Gómez S.S.A, Mexico City, Mexico; ^7^Department of Medicine, Hematology-Oncology Division, Greater Los Angeles VA Healthcare Center, UCLA Medical Center, Jonsson Comprehensive Cancer Center, Los Angeles, CA, United States

**Keywords:** miR-7, KLF4, YY1, hematological malignances, non-Hodgkin lymphoma

## Abstract

The discovery and description of the role of microRNAs has become very important, specifically due to their participation in the regulation of proteins and transcription factors involved in the development of cancer. microRNA-7 (miR-7) has been described as a negative regulator of several proteins involved in cancer, such as YY1 and KLF4. We have recently reported that YY1 and KLF4 play a role in non-Hodgkin lymphoma (NHL) and that the expression of KLF4 is regulated by YY1. Therefore, in this study we analyzed the role of miR-7 in NHL through the negative regulation of YY1 and KLF4. qRT-PCR showed that there is an inverse expression of miR-7 in relation to the expression of YY1 and KLF4 in B-NHL cell lines. The possible regulation of YY1 and KLF4 by miR-7 was analyzed using the constitutive expression or inhibition of miR-7, as well as using reporter plasmids containing the 3 ‘UTR region of YY1 or KLF4. The role of miR-7 in NHL, through the negative regulation of YY1 and KLF4 was determined by chemoresistance and migration assays. We corroborated our results in cell lines, in a TMA from NHL patients including DLBCL and follicular lymphoma subtypes, in where we analyzed miR-7 by ISH and YY1 and KLF4 using IHC. All tumors expressing miR-7 showed a negative correlation with YY1 and KLF4 expression. In addition, expression of miR-7 was analyzed using the GEO Database; miR-7 downregulated expression was associated with pour overall-survival. Our results show for the first time that miR-7 is implicate in the cell migration and chemoresistance in NHL, through the negative regulation of YY1 and KLF4. That also support the evidence that YY1 and KLF4 can be a potential therapeutic target in NHL.

## Introduction

Non-Hodgkin lymphomas (NHL) represent a heterogenous group of cancers that emerge from the monoclonal expansion of B or T lymphocytes; more than 90% of NHL are of B lymphocyte origin. The causes of NHL are poorly understood, and its pathogenesis is complex, and some subtypes are associated with several infectious agents. The different subtypes of lymphomas are consistent with the malignant clonal expansion of lymphocytes at different stages of differentiation. Because of this, the identification of different proteins, transcription factors and microRNAs (miRNAs) that participate in the control of the development of lymphoid progenitors have been of great interest, due to their possible association with various malignant processes in B-lymphocytes, including lymphomas, with potential implications for therapeutics and/or prognosis. Recent research has established miRNAs as predictors of chemo-sensitivity in cancer, as well as playing a role in reversing chemo-resistance in cancer cells (1). The presence of miRNAs in different hematological malignancies has been related to oncogenic characteristics or tumor suppressors, and many of them show dual activities that depend on cell type and cellular context (2). Several reports supports the argument that miRNAs act directly as tumor suppressors or promoters in hematological malignancies, since deregulation has been found in various cancers (3), including chronic lymphocytic leukemia (CLL), myeloma, and lymphoma (4). Several studies have demonstrated the presence of differential expression patterns of miRNA discriminating between normal and cancer cells, as well as discriminating between tumor sub-classification (5, 6). These patterns have also been identified in NHL and their sub-classifications; for example, an increase in the expression of miR-155 has been noted in aggressive DLBCL (7) and in NK lymphomas (8). This suggests that miR-155 plays a role in early oncogenesis, rather than secondary events culminating in the malignant process (9). Studies have established patterns of miRNAs expression that can predict the response to conventional treatment, as well as recurrence and survival of patients with DLBCL. Some of these miRNAs are miR-23a, miR-27a, miR-19a, miR-21, miR-92, miR-222, and miR-142 (10). Interestingly, miR-7 was identified as a possible predictor of response in follicular lymphoma, but its involvement in lymphomagenesis has not been studied (11). MiR-7 has been reported to be able to regulate the expression of KLF4 in breast cancer (12), and interestingly, in colon cancer it is able to inhibit the expression of YY1 (13), which suggests that miR-7 may have anti-tumor function. Recent studies by our group have shown that the expression of the KLF4 in lymphoma plays a pro-tumoral role and that it correlates with malignancy (14). Likewise, our working group and other groups have reported that the transcription factor YY1 is expressed in high levels in lymphomas, and we recently reported that this transcription factor regulates the expression of KLF4 in lymphoma cell lines (15). Because of this, in this study, we analyzed the possible role of miR-7 in lymphomagenesis by regulating YY1 and KLF4 in NHL. The participation of miR-7 in the pathogenesis of lymphoma, including its role in the regulation of transcription factors that establish more aggressive patterns of lymphoma, such as KLF4 and YY1, as well as its importance in lymphoma, has not been defined. Therefore, we hypothesized that miR-7 can play a role as tumor suppressor, through negative regulation of YY1 and KLF4 expression, affecting the cell migration and chemoresistance in NHL.

This hypothesis was tested by various means. 1) real-time RT-PCR analysis to determinate the expression of miR-7, KLF4, and YY1. In addition, western blot for KLF4 and YY1 in LNH cell lines. 2) B-NHL cell lines were transfected with miR-7 precursors and the overexpression of miR-7 was evaluated, as well as the inhibition by miR-7 inhibitor transfection, evaluating their role on the expression of KLF4 and YY1 in these cells lines and by using reporter plasmids containing the 3’UTR regions of KLF4 and YY1. 3) Cell viability and proliferation assays were performed on cell lines with overexpression or low expression of miR-7. 4) The malignant features of two B-NHL cell lines expressing opposite amounts of miR-7 or by constitutive expression plasmids and through chemoresistance and migration assays were analyzed. 5) Finally, in a tissue microarray (TMA) that includes 43 biopsies of patients with NHL, the expression of miR-7 was determined by ISH and YY1/KLF4 expression by Immunohistochemistry, and in GEO database analysis, miR-7 was analyzed in DLBCL and FL data.

## Materials and Methods

### Cell Culture

Ramos, Raji, DHL4, and 2F7, B-NHL cell lines were obtained from the American Type Culture Collection (ATCC, Manassas, VA), and cultured in an incubator at 37°C with 5% CO_2_ in RPMI 1640 Advanced medium (Gibco), supplemented with 4% Bovine Fetal Serum (SFB) (Gibco).

### RNA Extraction and Retro-Transcription

RNA extraction of 2x10^6^ cells from each of the cell lines was performed using the MiRneasy Thermo Fisher Kit, following the manufacturer instructions. Once the mRNA was obtained, retro-transcription was carried out using the “MultiScribe Transcriptase Reverse” kit from Thermo Fisher, to obtain the cDNA of each cell line, which was stored at -80°C.

### Real Time-PCR

Presence of miR-7 and the mRNA of KLF4 and YY1 was determined from total cDNA extracts obtained by miRneasy kit. For RT-PCR, Universal Taqman Master Mix II kit (Applied Biosystems) was used. Taqman probes and Taqman Universal Master Mix were used, and U6 was used as control of miR-7 expression, while for KLF4 and YY1, GAPDH transcript was evaluated. The relative value of expression of the genes and of the miRNA was calculated using the method 2^-(ΔΔCt)^ comparing the expression of mRNA of YY1 and KLF4 related to a GAPDH, and as an endogenous control for miR-7 we used U6.

### Western Blot

Cell lines were cultured at a density of 1x10^6^ cells/well in 2 ml of culture medium in 6-well cell culture plates, lysed with M-Per buffer (Thermofisher) and cell lysates were quantified with multi-reader EnSpire (PerkinElmer Massachusetts USA) using the Lowry method and subsequently denatured to 96°C, for 10 min. An electrophoretic shift was performed on an acrylamide gel, then transferred to a nitrocellulose membrane using a trans-Blot turbo (Bio-rad). The membranes were blocked for 1 h under constant agitation, primary antibodies directed to proteins Rabbit KLF4 (Novus Biologicals NBP1-83940 1:500) or YY1 (Novus Biologicals NBP2-67391 1:500), and GAPDH (Genetex 1:500) and were incubated overnight in constant agitation at 4°C and dark. Finally, 3 washes were performed with shaking with TBS-Tween 20 0.1% (5 min) and incubated with the secondary antibody (IRDye(R), 680RD [RED]. Donkey anti rabbit IgG Secondary antibody (P/N: 926-68073), IRDye(R), 800CW [GREEN]. Goat anti rabbit IgG Secondary antibody (P/N: 926-32211) LI-COR Biosciences), for 1 h and revealed with Oddissey Clx LI-COR equipment. The bands were detected by fluorescence using an Odyssey Clx reader (LI-COR, Lincoln, NE). The software Image Studio Lit Ver 5.2 was used to determinate the protein expression by counting the positive pixels.

### Patient Samples

Forty-three surgical specimens from biopsies of patients diagnosed with NHL were included (26 Follicular and 17 DLBCL samples). Samples were obtained from the Department of Pathology of the Oncology Hospital, CMN S.XXI, IMSS, and the General Hospital of Mexico Eduardo Liceaga SSA.

### Tissue Microarray (TMAs)

Biopsies from patients diagnosed with NHL were fixed in triplicate in a tissue microarray. Hematoxylin/Eosin (H&E) stains were made for an expert pathologist to select areas representative of the tumor, as previously reported, and new slides from same TMA previously reported were used in this work (15). Same samples of patients are included, but new stains and images were made for this study.

### *In Situ* Hybridization

For *in situ* hybridization, the XISH One step polymer-HRP Detection System for Xmatrx kit was used and the supplier’s instructions were followed. Hybridization was performed in the automated Xmatrx (Biogenex CA USA) using a lamella with the previously constructed microarray. Once the hybridization was completed, the slide was reviewed for digital pathology in the ScanScope equipment of Aperio (Aperio, Leica Biosystems, Germany), which has an analysis software micro tissue arrays in which you can determine the intensity of the expression by counting pixels in an automated way with which it was possible to determine the presence of the miR-7.

### Immunohistochemistry (IHC) and Morphometric Analysis

IHC stains were made as previously reported (15). Briefly, serial sections of the 3 μm thick from the TMAs were incubated overnight at room temperature with antibodies against KLF4 (Novus Biologicals NBP1-83940 1:500) or YY1 (Novus Biologicals NBP2-67391 1:500). The next day, the slides were washed and incubated with a second biotinylated antibody, part of a GBI kit (Golden Bridge International Labs.), for 15 min at room temperature, followed by an incubation with streptavidin conjugated to horseradish peroxidase (HRP) of the same kit (Golden Bridge International Labs.), for 15 min at room temperature. Subsequently, visible color generation was performed, 3,3’-tetrahydrochloride diaminobenzidine (DAB, GBI) was used from 1 to 5 min.

Immunohistochemically stained sections were digitized at a 40× magnification using an Aperio ScanScope CS (Aperio, Leica Biosystems, Germany). The Aperio ScanScope CS obtains 40× images with a spatial resolution of 0.45 μm/pixels. The images were reviewed using an ImageScope (Aperio, Leica Biosystems, Germany). Once the areas were annotated, they were sent for automated image analysis using Spectrum Software (Aperio, Leica Biosystems, Germany). For tissue intensity, an algorithm was developed to quantify the total miR-7, YY1, and KLF4 expression. The output from the algorithm returns a number of quantitative measurements, namely, the intensity, concentration and percentage of positive staining. Quantitative scales of intensity and percentage were categorized into 4 and 5 classes, respectively, after the cut-off values were determined in the three cores (spots) for each patient included in the TMA. The intensity of staining was categorized as 0 (no staining), 2+ (moderate), or 3+ (strong). The final IHC score was calculated from a combination of the intensity and percentage scores. Data are presented as total density/μm^2^ analyzed in a total area of 10,000 µm^2^.

### MTT Assay

50,000 cells of each cell line were seeded, incubated for 24 h with 330 µM of cis-diaminedichloroplatine (II) (CDDP), and then the MTT reagent was added, after 4 h the solubilization of the salts was carried out by means of the solubilizing agent included in the MTT kit to later incubate for one night. Once the incubation occurred, the plate is revealed by quantifying the absorbance in a multi-reader EnSpire (PerkinElmer Massachusetts USA) plate of PE at 620 nm.

### Migration Analysis

Transwell chambers were used. Membrane was activated with the addition of 500 microliters of RMPI 1640 Advanced culture medium, without fetal bovine serum for 1 h at 37°C. After 1 h, the medium was added with 0.5x10^6^ cells on the membrane and circular movements were made for an adequate distribution. RPMI 1640 Advanced culture medium with 5% and 10% FBS was added to the six wells plate with 6µM diameter (Costar, ME. USA). It was incubated at 37°C with 5% CO2 for 24 h and the number of cells was determined on the membrane and in the culture, medium placed under the membrane, by staining with trypan blue in an automated counter of TCD10 cells (BioRad CA USA).

### Reporter, Inhibitor, and Mimetizer of miR-7 Transfection

The “Lightswitch miRNA mimics and inhibitors” system of SwitchGearGenomics was used, which consists of an inhibitor of miR-7 that allows a “knock down” of the miRNA by complementarity, as well as a mimetizer that allows generating a sequence identical to miR-7 that allows the synthetic increase in miRNA expression. A reporter that contains the 3’UTR region of KLF4 and YY1 separately followed by a luciferase reporter were used. In all cases, 1 μg of the reporter and 2 μg of the mimetic or inhibitor were transfected. They were incubated for 24 h and subsequently the luminescence was determined with a multi-plate reader enSpire EnSpire (PerkinElmer Massachusetts USA) for detection of luminescence.

### Transfection of B-NHL Cell Lines

2.5 X 10^5^ cells were cultured in 12-well plates, 700 microliters of RPMI culture medium without supplementation, then the lipofectamine mixture was added with the genetic material and incubated for 5 h at 37^0^C, after this time the medium was changed by means of supplemented and incubated for 48 h at 37°C. Finally, the luminescence was determined by addition of the luminescence substrate in an enSpire plate multi-reader (PerkinElmer Massachusetts USA).

### Immunofluorescence

Rabbit anti-YY1 (Novus Biologicals NBP2-67391 1:250), Rabbit anti-KLF4 (Novus Biologicals NBP1-83940 1:250), anti-rabbit IgG Isotype control, AlexaFluor 488 Streptavidin (Jackson-lmmunoresearch, West Grove, CA, USA), and Vectashield- DAPI (Vector-laboratories, Burlingame, CA, USA) were used to stain Ramos cell lines transfected with INH or MIM-miR-7. Images were acquired using a Leica TCS SP8x Confocal Microscope (Wetzlar, Germany) and were analyzed with Leica software.

### Data Source and miRNA Expression Analysis

miRNA expression in lymphoma (147 lymphoma tissues and 7 normal tonsillar tissues, GSE29493), (29 DLBCL, 23 FL, and 4 normal GC-B cells GSE29493) (16), (7 DLBCL dead patients and 7 DLBCL alive patients for overall survival, all patients receiving R-CHOP treatment GSE10846) (17), were obtained from the Gene Expression Omnibus (GEO, https://www.ncbi.nlm.nih.gov/geo/). All GEO data were analyzed by GEO2R or R. Overall Survival distributions for miR-7 was estimated by the Kaplan-Meier method, with differences evaluated by the log-rank test. OS is defined as the time from initial diagnosis to death or last follow-up, with those alive at last follow-up treated as censored.

## Results

### miR-7 Expression Correlates Negatively With the Expression of KLF4 and YY1 in B-NHL Cell Lines

**Figure 1:**
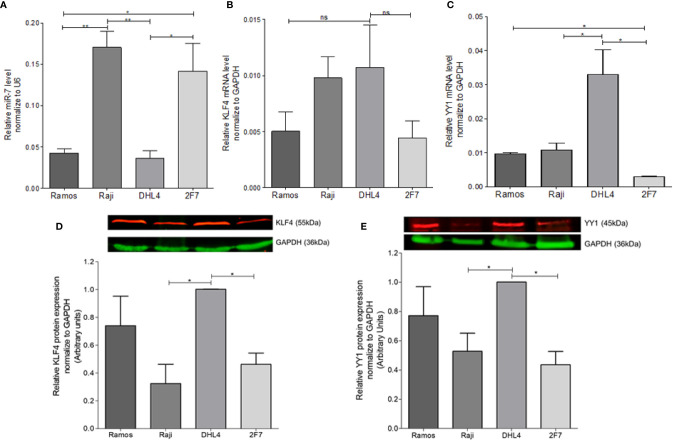
Determination of miR-7, KLF4, and YY1 expression in lymphoma cell lines. **(A)** miR-7 expression in B non-Hodgkin lymphoma (B-NHL) cell lines was determinate by real time PCR. The values were calculated by the ΔΔCt method and was evaluated, endogenous control U6 was used as a control. Three-triplicated independents experiments were done and evaluate the relative level, statistical differences are shown (*p < 0.05 **p < 0.01). **(B, C)** KLF4 and YY1 mRNA expression was determinate by Real Time PCR using GAPDH as an endogenous control. Three-triplicated independents experiments were done and evaluate the relative level, statistical differences are shown. YY1 relative levels were significant (*p < 0.05). **(D, E)** Representative images of protein expression of KLF4 and YY1 respectively, evaluated by western Blot. The red lines represent the protein expression and in Green the endogenous control (GAPDH). Three independent experiments were done and evaluate the pixel densitometry (arbitrary units) Bottom plots, *p < 0.05.

In order to determine the possible correlation of miR-7 expression with the expression of YY1 and KLF4 in different B-LNH cell lines, real-time PCR was performed. miR-7, YY1, and KLF4 expression was analyzed in four B-NHL cell lines: Ramos, Raji, DHL4 and 2F7. The results shown that the Raji and 2F7 cell lines have a higher expression of miR-7 as compared with Ramos and DHL4 cell lines. (**p<0.01, *p<0.05) (**Figure 1A**). Analysis of KLF4 mRNA expression showed that DHL4 has a higher KLF4 (**Figure 1B**) and YY1 (**Figure 1C**) expression than the other NHL cell lines analyzed (*p<0.05). YY1 and KLF4 mRNA expression versus miR-7 levels, show that, there is no significant inverse correlation (data no shown). This result allowed us to select two cell lines, one with high miR-7 expression (Raji), and the other with low miR-7 expression (DHL4), for further studies.

In previous studies we showed that there was a correlation between YY1 and KLF4 proteins in B-NHL cell lines. To corroborate prior results on YY1 and KLF4 mRNA expression, KLF4 and YY1 protein expression was analyzed by western blot, and the intensity of expression was determined by densitometry (**Figures 1D, E****)**. The results show a high YY1 expression in the Ramos and DHL4 cell lines as compared with Raji and 2F7 cell lines, showing a statistically significant difference for Raji vs DHL4 (*p<0.05) (**Figure 1E**). Similar results were observed for the expression of KLF4, where the Raji and 2F7 cell lines have a lower expression compared to Ramos and DHL4 (*p<0.05) (**Figure 1D**). As mentioned, Raji and DHL4 cell lines showed a greater difference in the expression of miR-7, as well as KLF4 and YY1, so they were selected for subsequent experiments. These results show that there is an inverse correlation in the expression of miR-7 and the transcription factors YY1 and KLF4.

### miR-7 Regulate the Expression of YY1 and KLF4 by Binding to a 3`UTR

**Figure 2:**
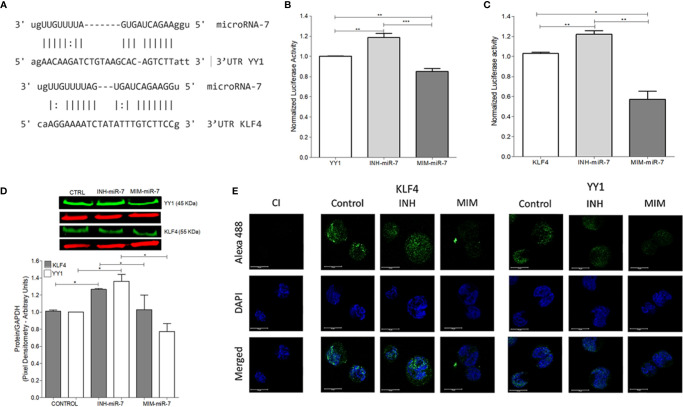
miR-7 regulate the expression of YY1 and KLF4 by binding its 3`UTR region. **(A)** Analysis of miR-7 binding sites in the 3`UTR regions of KLF4 and YY1, through the miRtarbase database. **(B)** Raji cell line was previously co-transfected with an inhibitor (INH) or a mimic (MIM) of miR-7, and then transfected with the reporter plasmid with the luciferase gene containing the 3 ‘UTR region of YY1 **(B)** and KLF4 **(C)**. The presence of INH for miR-7 increases the expression of KLF4 and YY1 (***p < 0.001) and the MIM inhibits its expression (**p < 0.001 *p < 0.05), respectively. **(D)** Left: Western blot for KLF4 and YY1 was done, after treatment with an inhibitor or miR-7 mimic compared to a control. Right: densitometric analysis of YY1 and KLF4 expression was done and significant differences were indicated (*p < 0.05). **(E)** Immunofluorescence in Raji cell lines was done and expression of KLF4 and YY1 were evaluated after transfection with an inhibitor or miR-7 mimic. Representative images of a triplicate of each experiment are shown.

As already mentioned, previous studies have reported that miR-7 negatively regulates the expression of YY1 (13) and KLF4 (12). In this study, we analyzed whether there are possible binding sites of miR-7 in the 3’UTR regions of KLF4 and YY1 through the miRtarbase database (mirtarbase.mbc.nctu.edu.tw) (18), finding at least one binding site in each of the 3’UTR regions of the transcription factors (**Figure 2A**). The ability of miR-7 to transcriptionally regulate YY1 and KLF4 was evaluated by reporter plasmid assays, and with the upregulation or downregulation of miR-7 expression by a mimic-miR-7 (MIM-miR-7) or an inhibitor-miR-7 (INH-miR-7), respectively. Plasmids containing luciferase and the 3`UTR region of KLF4 and YY1 were used as reporter. The results show that the miR-7 mimic decreased the reporter’s signal for both YY1 (**Figure 2B**) and KLF4 (**Figure 2C**), (*p<0.05**p<0.01 ***p<0.001), while the miR-7 inhibitor was able to increase the expression of YY1 and KLF4 (**Figures 2B, C****)**. These results allow us to conclude that miR-7 can regulate the expression of KLF4 and YY1 by binding to its 3`UTR region.

To evaluate the effect of the inhibitor and mimic of miR-7 *in vitro*, we perform a western blot to evaluate protein expression for both transcription factors. The results show that the mimic decreased expression of both transcription factors, while transfection with the miR-7 inhibitor induced higher expression, compared with untransfected control (*p<0.05). These results support the role of miR-7 in the regulation of YY1 and KLF4. (**Figure 2D**). To validate the role of miR-7 in the transcriptional regulation of YY1 and KLF4, we performed immunofluorescence to detect the expression of both transcription factor in the cells transfected with the inhibitor or mimic of miR-7 and an untransfected control. We used DAPI to contrast DNA. The results are consistent with the western blot analysis: we observe a decrease in the KLF4 and YY1 expression in the case of the miR-7 mimic (MIM), and increased expression of both transcription factors in the cells with the miR-7 inhibitor (INH) (**Figure 2E**). Together these results confirm that miR-7 negative regulated YY1 and KLF4.

### miR-7 Is Involved in Migration and Chemoresistance of B-NHL Cell Lines

**Figure 3:**
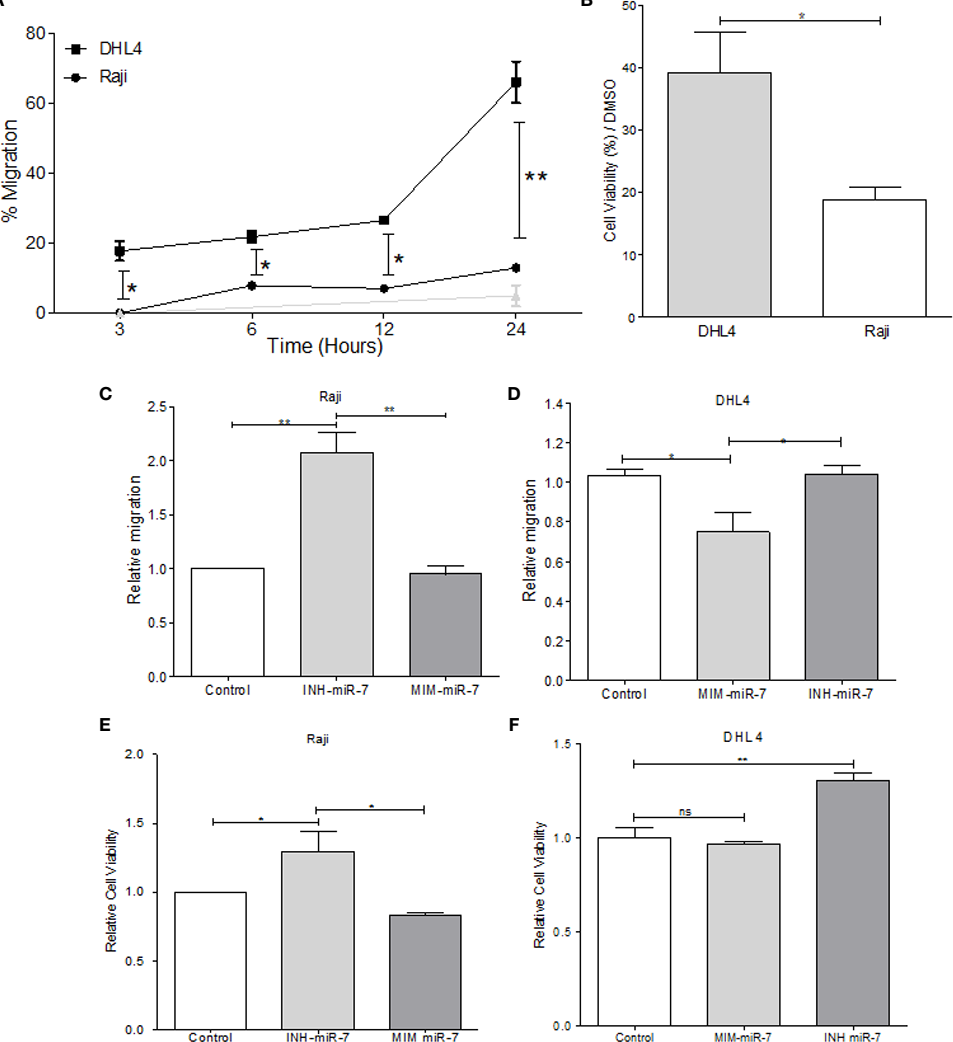
miR-7 play a role in the migration and chemoresistance in non-Hodgkin lymphoma (NHL) cell lines. **(A)** migration assay in Transwell™ chambers. Percent of total cell numbers was determinate in Raji and DHL4 cells at 3, 6, 12, and 24 h. (*p < 0.05, **p < 0.001). **(B)** Chemoresistance was evaluated by cellular viability after CDDP treatment (330 µM). Percent of viability was reported for Raji and DHL4 cell line (*p < 0.05). **(C, D)** Migration assay in the Raji cell line transfected with INH-miR-7 or MIM-miR-7 and DHL4 cells transfected with the MIM-miR-7 or INH-miR-7 as well, respectively (*p < 0.05) was done. **(E, F)** Chemoresistance in Raji cell line transfected with the INH-miR-7 or MIM-miR-7 and DHL4 cells transfected with the MIM-miR-7 or INH-miR-7, respectively (*p < 0.05) was done. Results of three independent experiments are shown.

In order to evaluate the role of miR-7 in NHL, B-NHL cell lines with higher or lower miR-7 expression (Raji and DHL4 respectively) were selected. Migration assays were carried out in Transwell™ chambers. Results shown that DHL4 cell line has a higher migration rate in relation to the Raji cell line (**p< 0.01 *p<0.05), (**Figure 3A**). This suggests that the low miR-7 expression, and its inverse correlation with the high YY1 and KLF4 expression, could be related to a higher migration capacity. Another of the characteristics evaluated was chemoresistance, which was assessed by inhibition of proliferation, determined by MTT assay. Raji and DHL4 cell lines were treated with a chemotherapeutic agent cis-diaminedichloroplatine (II) (CDDP). The results show that the Raji cell line has a low proliferation and cell viability, compared with DHL4 after CDDP treatment compared to the control (*p<0.05), (**Figure 3B**), which indicates that the DHL4 cells, which have low levels of miR-7 expression and high levels of YY1 and KLF4, show greater chemoresistance, suggesting a possible role for miR-7 in chemosensitivity, through the negative regulation of YY1 and KLF4.

To corroborate the role of miR-7 in the migration and chemoresistance of B-NHL cell lines, we transfected the Raji cell line that shows a high expression of miR-7 with an inhibitor of miR-7 (INH-miR-7), or mimic-miR-7 (MIM-miR-7) respectively. After the transfection, a migration assay was done (**Figure 3C**). The results show that the migration capacity of Raji cells transfected with the miR-7 inhibitor increased more than two times, compared to the cells transfected with a negative control or MIM-miR-7 (*p<0.05). In addition, we transfected the DHL4 cell line that has low miR-7 expression with MIM-miR-7 or INH-miR-7 respectively, where only transfection with MIM-miR-7 resulted in inhibition of migration capacity (*p<0.05) (**Figure 3D**).

These results confirm a role of miR-7 in the migration capacity of B-NHL cell lines. Chemoresistance was also analyzed in Raji-INH-miR-7, Raji-MIM-miR-7, DHL4-MIM-miR-7, and DHL4-INH-miR-7 (**Figures 3E, F****)**. The results shown that miR-7 inhibition on Raji cell line induced chemoresistance compared to non-transfected cells or MIM-miR-7 (*p<0.05) **(****Figure 3E****)**. CDDP treatment of DHL4-MIM-miR-7, results in a non-altered cell viability compared with the control or INH-miR-7, However, the use of INH-miR-7 increases chemoresistance of DHL4. (**Figure 3F**). Together this confirms that miR-7 plays a role in the migration and chemoresistance in B-NHL cell lines.

### miR-7 Expression Correlates Negatively With the Expression of KLF4 and YY1 in Biopsies From Patients With NHL

**Figure 4:**
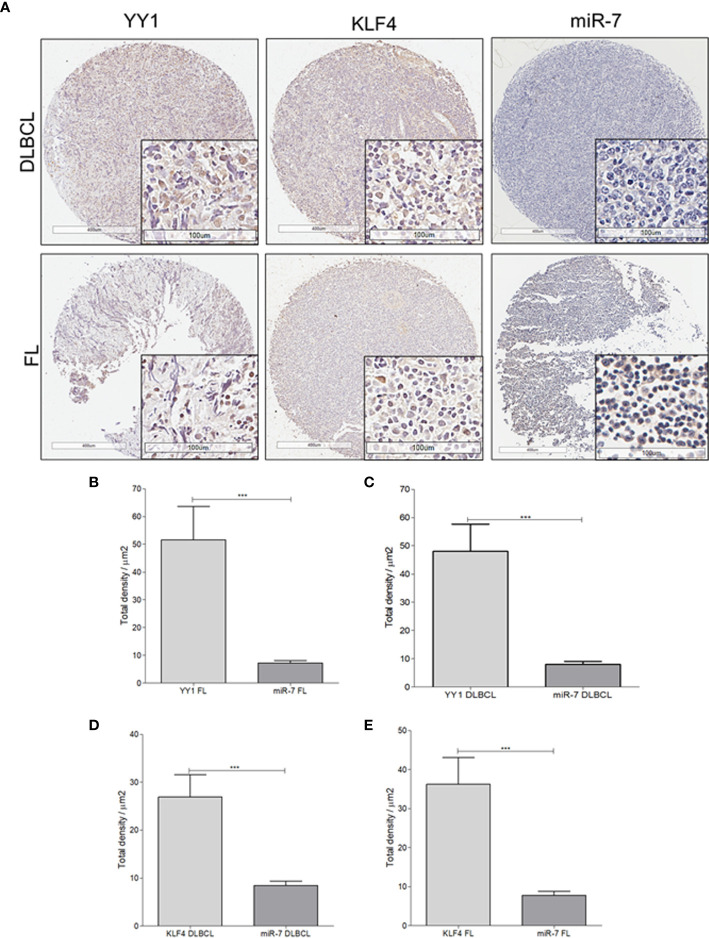
Expression of miR-7, KLF4, and YY1 in NHL tissues. **(A)** Representative micrographs of KLF4 and YY1 expression analyzed by immunohistochemistry, and miR-7 expression evaluated by *in situ* hybridization in a tissue microarray of patients with NHL are shown. (Top) different markers in biopsies of DLBCL. (Below) representative micrograph of different markers in follicular lymphoma biopsies (original magnification: 4X and 40X in the frame. **(B–E)** Density expression of miR-7, YY1, and KLF4 were reported and total expressions for each subtype were compared (***p < 0.001).

To extend our findings from B-NHL cell lines, miR-7 expression was analyzed by *in situ* hybridization, and YY1 and KLF4 were analyzed by immunohistochemistry, in an NHL TMA (**Figure 4A**). Expression was analyzed by digital pathology; the results show that there a significant inverse correlation between miR-7 and YY1 and KLF4 expression (***p<0.001) (**Figures 4B–E**).

**Figure 5:**
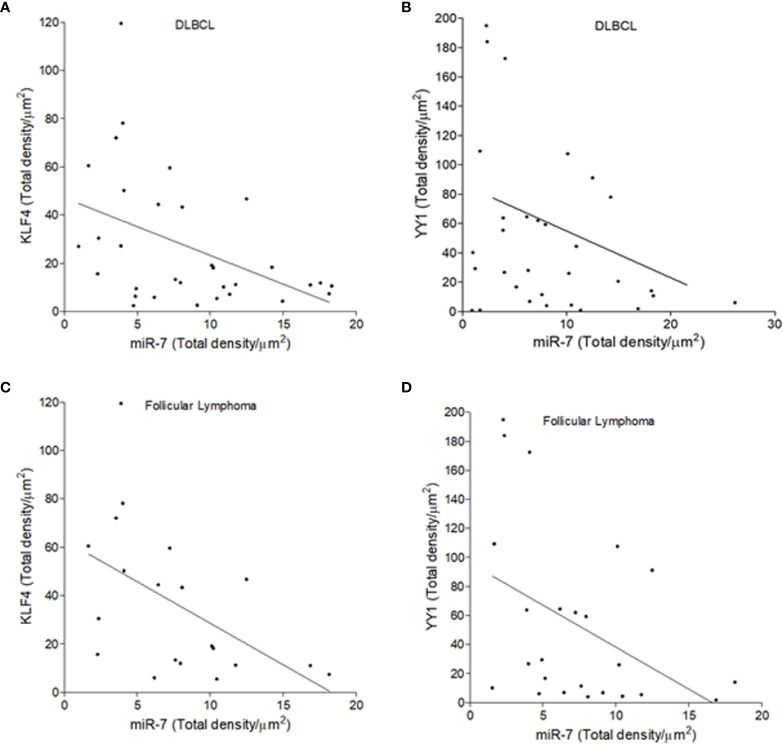
Inverse expression correlation of miR-7 vs YY1/KLF4 in non-Hodgkin lymphoma (NHL) tissues. **(A–D)** Correlation of the expression of YY1 vs miR-7, or KLF4 vs miR-7 in DLBCL and follicular lymphoma was analyzed, respectively. Expression correlation analysis of miR-7 vs KLF4/YY1 was evaluated by Pearson correlation in DLBCL **(A)** (r=-0.4454 p=0.0106) and **(B)** (r=-0.3415 p=.0301) or follicular lymphoma **(C)** (r=-0.5229 p=0.0180) and **(D)** (r=-0.4248 p=0.0385).

To establish if there is an inverse correlation in the expression of miR-7 vs that of YY1/KLF4 in biopsies from patients with NHL, a statistical analysis of Pearson’s R regression was performed. The results obtained for DLBCL (**Figures 5A, B****)** show a significant negative correlation of KLF4 vs miR-7 (r=-0.4454, *p*=0.0106), as well as for YY1 *vs* miR-7 (r=-0.3415, *p*=0.0301). The results for follicular lymphoma (**Figures 5C, D****)** show that KLF4 vs miR-7 have a negative correlation (*r*=-0.5229, *p*=0.0180), and miR-7 *vs* YY1 also have a negative correlation (*r*=-0.4248, *p*=0.0385). Therefore, our results strongly suggest that there is a significant negative correlation between miR-7 expression and that of YY1 or KLF4 in DLBCL and follicular lymphoma (**Figure 5****)**.

### Bioinformatics Analysis of miR-7 in Lymphoma

**Figure 6:**
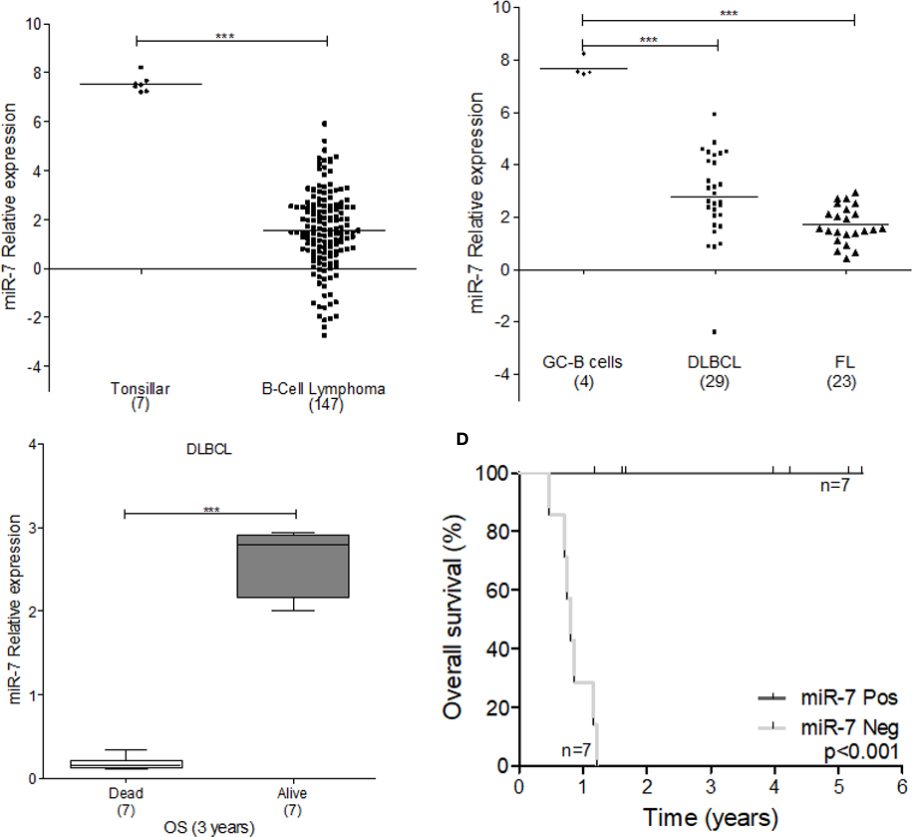
Expression of miR-7 is downregulated in lymphoma tissues. **(A)** Expression of miR-7 in B-Cell lymphoma is downregulated compared to normal tonsillar tissue (***P < 0.001), **(B)** miR-7 expression was downregulated in DLBLC and FL compared to normal GC B-cells (***P < 0.001). Data set obtained from GEO: GSE29493 **(C)** miR-7 expression is downregulated in dead patients compared to alive patients attending to overall survival at 3 years. Data set obtained from GEO: GSE31312 (***P < 0.001). **(D)** Overall survival of patients with diffuse large B-cell lymphoma according to miR-7 expression. The number of patients (n) is listed next to the result (***p < 0.001).

To explore the tumor promoting or suppressive effects of miR-7 in lymphoma, we used microarray analysis data from GEO datasets. Our results show that miR-7 expression is significantly downregulated in B-cell lymphoma compared to normal tonsillar tissues used as a control (***p<0.001) **(****Figure 6A**). Additionally, we compared miR-7 expression in lymphoma tissues from DLBCL and FL subtypes to that seen in germinal center (GC) B-cells (***p<0.001 *p<0.05) (**Figure 6B**), finding higher levels of miR-7 expression in normal GC B cells. In addition, we analyzed if tumor cell miR-7 expression can correlate with overall survival (OS) of lymphoma patients. Results suggested, that high miR-7 expression correlated with better OS in DLBCL patients (***p<0.001) (**Figures 6C, D**).

## Discussion

Several studies have shown that miRNAs play important roles in different biological processes, such as proliferation, cell death, cell cycle, and in cancer. The regulation of miRNA expression has been described as an important factor in the progression and initiation of cancer, for this reason these molecules have been implicated in the development of various malignancies (3, 5, 19). One of the miRNAs that has been associated with cancer is miR-7 (20). Specifically, it has been shown that it has the capacity negatively regulate the transcription factors KLF4 (12) and YY1 (13). KLF4 and YY1 have been directly related to a poor prognosis in patients diagnosed with NHL (14, 21). Therefore, in this study we evaluated the participation of miR-7 in the regulation of YY1 and KLF4 expression in B-NHL, and its possible role in the malignancy, mediated by regulating the expression of YY1 and KLF4. The results show an important inverse correlation of the expression of miR-7 and the transcription factor YY1 and KLF4 on B-NHL cell lines here analyzed. These results are consistent with recent studies (12, 13), and suggest that there is an inverse expression between miR-7 and YY1/KLF4, and this could be related to the transcriptional regulation of KLF4 and YY1 by miR-7 in B-NHL (15). We confirm these results using a reporter plasmid (luciferase) containing the 3 ‘UTR regions of both transcription factors and miR-7 inhibitor that decreases miR-7 expression or a miR-7 mimic that increases miR-7 expression. The results show that the miR-7 negatively regulates the expression of KLF4 and YY1 by binding to its 3’UTR region in B-NHL cell lines (**Figures 2A–C**). Then, we evaluated some the relationship of miR-7 expression with features of malignancy, such as cellular migration, as well as resistance to chemotherapy using CDDP (12, 22). Migration and chemoresistance assays showed that miR-7 was associated with a reduced migratory capacity and lower cell viability after CDDP treatment (**Figure 3**). In order to confirm this, B-NHL cell lines were transfected with a mimic or an inhibitor of miR-7 to subsequently evaluate migration and chemoresistance. As shown in **Figure 3C**, transfection with miR-7 inhibitor in the Raji cell line increases the migration capacity by almost two times. On DHL4 cell line that was transfected with the mimic shown to decrease migration capacity (**Figure 3D**). In addition, same previously transfected cell lines were used to evaluate chemoresistance (**Figures 3E, F****, respectively)**.

Results shows that miR-7 inhibition on Raji cell line induced chemoresistance, however, in DHL4, miR-7 mimic shows no difference in chemoresistance compared to the control this could be explained by the high levels of chemoresistance showed by DHL4 cell line to CDDP. However, the inhibition of miR-7 by INH-miR-7 increases the chemoresistance of DHL4, suggesting that miR-7 plays a role in the chemoresistance of the DHL4 cell line. Additionally, as we observe in **Figures 1D and E**, DHL4 cell line has the highest levels of YY1 and KLF4 protein expression this could provide an high chemoresistance probably by the role of YY1 in the regulation of MDR1 (23). Higher concentrations of CDDP and another drug needs to be evaluated. Also, these results support our proposition in which miR-7 could regulates the expression of YY1 and his role in the malignancy of B-NHL. Together the results indicate that miR-7 expression plays a role in the migration capacity and chemoresistance, by regulating specific targets such as YY1 and KLF4, where these have previously been reported by our working group and others, have a role in the malignancy of lymphoma (12, 13, 22).

YY1 is a zinc finger protein that is ubiquitously expressed and is involved in a large number of biological processes, such as development, proliferation, differentiation and apoptosis (24). Its specific function depends on its location and is determined by its network of interaction with other proteins, DNA, and RNA (25). YY1 is critical in the regulation of the early development of B lymphocytes (26). Additionally, it is critical in secondary lymphoid tissues, such as the lymph nodes, where the maturation and expansion of B lymphocytes occurs (27), and which is the site of origin of several NHL subtypes, such as DLBCL and follicular lymphoma. Therefore, YY1 has been implicated in several hematological malignancies, including leukemia and NHL (28). For this reason, YY1 has been proposed as an important biomarker and a potential therapeutic target in NHL (29). It has been reported that samples of DLBCL and FL show high levels of expression of YY1 and this correlates with a poor survival of patients (30). Additionally, YY1 *in vitro* can promote the transformation of B lymphocytes and contribute to tumor progression (21), or contribute to the chemoresistance of NHL (31).

KLF4 can activate or repress different genes (32), and in particular in pre-B lymphocytes, it plays an important role in the regulation of the cell cycle (33). Recent studies have reported that the expression of KLF4 correlates with poor survival and response to treatment in pediatric patients with Burkitt’s lymphoma (14), and it has also been described that KLF4 regulates the cell cycle in hematologic malignancies (34), and is expressed in leukemia and lymphoma cell lines. Interestingly, recent studies by our group have reported that YY1 is able to regulate the expression of KLF4 in B-NHL (15), which establishes a network of expression between YY1 and KLF4, at least in the case of NHL, and establishes its importance and its possible role in lymphomagenesis. Clearly, the evidence so far suggests that the YY1/KLF4 axis is an important biomarker with possible diagnostic and therapeutic uses. By using chemical inhibitors or siRNAs, as previously reported (14, 21)

The results reported here obtained with B-NHL cell lines were corroborated in biopsies of patients with NHL. In a tissue microarray (MAT) we analyzed miR-7, YY1 and KLF4 expression (**Figure 4**). Expression analysis indicate that there is a directly proportional correlation between KLF4 and YY1, while the relationship between miR-7 with both transcription factors has an inverse correlation (**Figure 5**). Recent publications have reported that there is a correlation of clinical parameters and the expression of miR-7 in follicular lymphomas (11). Also, we corroborated our findings in B-NHL cell lines, performed miR-7 expression evaluation in a microarray analysis data from GEO datasets. The results shown that miR-7 expression was downregulated in lymphoma tissues compared to tonsillar tissue (**Figure 6A**); the downregulation of miR-7 expression was clearly in DLBCL and FL subtypes compared to GC B-cells (**Figure 6B**).

FL is the most common of the indolent NHLs, and it is the second most common of NHL subtype (35). This subtype of lymphoma is incurable, although with new therapeutic alternatives, the possibility of survival at 5 years is around 70%. There are no established treatment protocols because FL is highly heterogeneous. However, the identification of new therapeutic targets with the possibility of improving response rates to treatment continues. Recently, miRNAs have been described as having important functions in the biology of lymphomas, including follicular lymphoma. In malignant B cells, miRNAs participate in pathways essential for the development of B cells such as the B cell receptor (BCR), migration/cell adhesion, B cell interactions with niches of the immune system, and the production and the isotope change of immunoglobulins (36). miRNAs influence the maturation, generation of pre-B cells, marginal zone, follicular cells, B1 cells, plasma cells, and memory B cells (37). Recently, a unique expression signature of microRNAs has been proposed, where expression pattern could be involved in cell survival and proliferation. Wang W et al. (11). performed an analysis of 851 microRNAs, of which they identified three groups with a clear expression difference (> 2 times, *p*<0.05). In their group 2, miR-7 was analyzed together with other miRNAs; miR-7, showed a direct correlation of high expression to complete response to treatment, while the low expression of miR-7 was associated with a poor response (11). These results are consistent with our bioinformatic GEO analysis (**Figures 6C, D**).

In a molecular and genetic approach, FL has genetic alterations that can be defined and characterized by their biological and clinical importance (38). Therefore, defining expression patterns of miR-7, not only in FL but in other subtypes of B-NHL, is of great interest. miRNAs can regulate multiple transcripts and a transcript can be under the control of several miRNAs, and their dysregulation can contribute to the pathogenesis of B-NHL, and these transcripts can be used as potential targets for diagnosis, prognosis and therapies evaluation. Recently, Getaneh, Z et al. (39), analyzed the different expression patterns of miRNAs in the most common B-NHL, assessing their possible role in pathogenesis and their potential with therapeutic implications (3, 39)

The results of this work show, for the first time, that two transcription factors, YY1 and KLF4, which are known to participate in the pathogenesis of NHL, and also have been proposed as targets for pharmacological inhibitors as a potential alternative treatment (15), are regulated by miR-7, which can modulate the capacity of NHL cells for proliferation, migration and chemoresistance. This result suggests that the miR-7/YY1/KLF4 axis can be a biomarker of malignancy in B- NHL. Additionally, as we mentioned before, KLF4 and YY1 have great potential to be targets in the treatment of NHL (14, 21).

This study also provides the bases that establish the suppressive role of miR-7 in NHL, providing a guideline for its study as a therapeutic target through miRNA replacement therapy, using the method of miRNAs mimicking nano-cells of the EDV ™ type, which has proved to be a great boon for its ability to restore miRNA function and re-sensitize chemotherapy resistant tumor cells. The fact that several miRNA replacement therapies are currently in a clinical trial demonstrates the great potential of this approach in treating cancer (40).

## Conclusion

**Figure 7:**
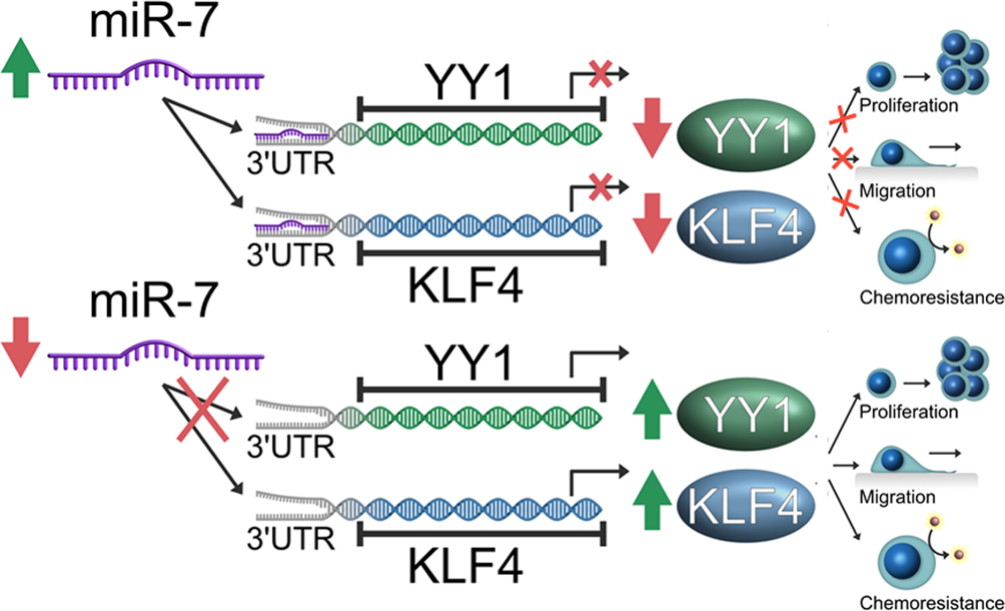
Schematic representation of the model of the role of miR-7 in the YY1/KLF4 axis regulation, and the implication of this axis in migration and chemoresistance of non-Hodgkin lymphoma (NHL). Our model proposes that miR-7 plays a role as tumor inhibitor in Lymphoma, where a low expression of miR-7 allows the high expression of YY1/KLF4 with the final result of the increase in proliferation, migration and chemoresistance, while high miR-7 expression inhibits YY1/KLF4 expression by attenuating proliferation and migration and reversing chemoresistance in NHL.

In conclusion, our findings demonstrate that miR-7 regulates transcriptional YY1 and KLF4 in NHL, and downregulation of miR-7 impacts the capacity of migration and chemoresistance *in vitro*. Inverse correlation expression was corroborated in samples of patients with NHL, which is consistent with the results obtained *in vitro* and suggested the participation of miR-7 in the axis YY1-KLF4, thus forming an important descriptive model in the pathogenesis of the disease (**Figure 7**).

## Data Availability Statement

The raw data supporting the conclusions of this article will be made available by the authors, without undue reservation.

## Ethics Statement

The studies involving human participants were reviewed and approved by Comité de Ética en Investigación CONBIOETICA-09-CEI-099-20160601, IMSS. Written informed consent for participation was not required for this study in accordance with the national legislation and the institutional requirements.

## Author Contributions

Performed the experiments and GEO analysis: MM-M. Analyzed the data and interpreted the results: MM-M, GV, NN, MN, IA, IC, MD-P, SH-Y, MV. Contributed valuable tumor tissue and clinical information: NN, MN, IA, IC, MD-P. Provided tumor tissues and performed pathologic analysis of specimens: IA, IC, MD-P. Designed, supervised, and/or obtained support for the study: SH-Y, MV. Wrote the manuscript: MM-M, MV. All authors contributed to the article and approved the submitted versión.

## Funding

This study was supported in part by grant FIS/IMSS/PROT/PRIO/14/035 from the IMSS (MV); UC MEXUS-CONACYT Collaborative Research Grants (CN-11-554 MV and Dr. Otoniel Martinez-Maza); and CONACyT (MV CB-2011-169368).

## Conflict of Interest

The authors declare that the research was conducted in the absence of any commercial or financial relationships that could be construed as a potential conflict of interest.

## Abbreviations

B-NHL, B non-Hodgkin lymphoma; DLBCL, diffuse large B cell lymphoma; FL, Follicular Lymphoma; IHC, immunohistochemistry; ISH, *in situ* Hybridation; KLF, Krüppel-like Factor; miR-7, MicroRNA-7; YY1, Yin Yang 1.
